# A Review on the Phytochemistry, Pharmacology, Pharmacokinetics and Toxicology of Geniposide, a Natural Product

**DOI:** 10.3390/molecules22101689

**Published:** 2017-10-10

**Authors:** Mingqiu Shan, Sheng Yu, Hui Yan, Sheng Guo, Wei Xiao, Zhenzhong Wang, Li Zhang, Anwei Ding, Qinan Wu, Sam Fong Yau Li

**Affiliations:** 1 Jiangsu Collaborative Innovation Center of Chinese Medicinal Resources Industrialization, Nanjing University of Chinese Medicine, Nanjing 210023, China; yusheng1219@163.com (S.Y.); glory-yan@163.com (H.Y.); gsh916@163.com (S.G.); zhangliguanxiong@163.com (L.Z.); awding105@163.com (A.D.); qnwyjs@163.com (Q.W.); 2 National Key Laboratory of Pharmaceutical New Technology for Chinese Medicine, Jiangsu Kanion Pharmaceutical Co. Ltd., Lianyungang 222001, China; xw_kanion@163.com (W.X.); wzhzh-nj@163.com (Z.W.); 3 Department of Chemistry, National University of Singapore, Singapore 117543, Singapore; chmlifys@nus.edu.sg

**Keywords:** geniposide, iridoid glycoside, natural product, pharmacokinetics, pharmacology, phytochemistry, toxicology

## Abstract

Iridoid glycosides are natural products occurring widely in many herbal plants. Geniposide (C_17_H_24_O_10_) is a well-known one, present in nearly 40 species belonging to various families, especially the Rubiaceae. Along with this herbal component, dozens of its natural derivatives have also been isolated and characterized by researchers. Furthermore, a large body of pharmacological evidence has proved the various biological activities of geniposide, such as anti-inflammatory, anti-oxidative, anti-diabetic, neuroprotective, hepatoprotective, cholagogic effects and so on. However, there have been some research articles on its toxicity in recent years. Therefore, this review paper aims to provide the researchers with a comprehensive profile of geniposide on its phytochemistry, pharmacology, pharmacokinetics and toxicology in order to highlight some present issues and future perspectives as well as to help us develop and utilize this iridoid glycoside more efficiently and safely.

## 1. Introduction

**Figure 1:**
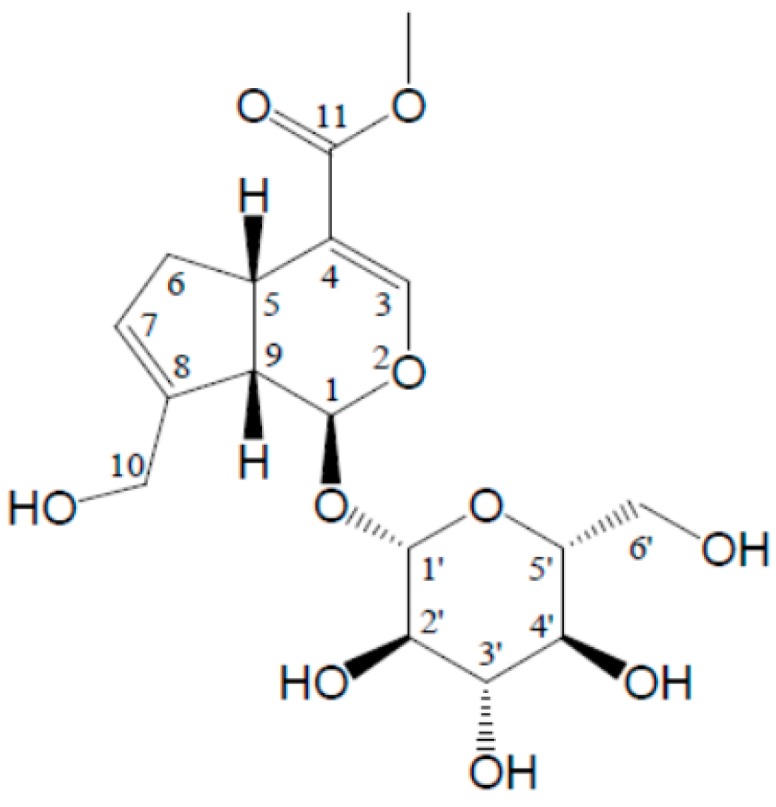
Chemical structure of geniposide.

Iridoid glycosides are phytochemicals which naturally occur in many plants belonging to the families Scrophulariaceae, Rubiaceae, Gentianaceae and Caprifoliaceae, including some Traditional Chinese Medicines (TCMs). Among them, geniposide (methyl (1*S*,4*aS*,7*aS*)-1-(β-d-gluco-pyranosyloxy)-7-(hydroxylmethyl)-1,4*a*,5,7*a*-tetrahydrocyclopenta[c]pyran-4-carboxylate; C_17_H_24_O_10_; GS; Figure 1) is particularly well known. In view of its chemical structure, this component is also considered as a glycoside consisting of one molecule each of genipin and glucose. To date, this natural product has been isolated and identified in nearly 40 plants, most of which are traditional phytomedicines and/or come from the family Rubiaceae. As far as bioactivities are concerned, GS exerts many pharmacological functions, including anti-inflammatory [1], antidiabetic [2], anti-oxidative [3], neuroprotective [4], hepatoprotective and choleretic effects [5]. GS is recorded as a characteristic component for the quality control of *Gardenia jasminoides* Ellis (*G. jasminoides*) fruits in the 2000–2015 editions of the Chinese Pharmacopoeia [6,7,8,9]. According to the national standard, this phytomedicine with a GS content above 1.8% could be considered as having adequate quality. Additionally, in the 2015 edition, GS as also listed as the quantitative component for quality evaluation of about twenty Chinese Patent Medicine preparations containing *G. jasminoides* fruits, such as Bazheng mixture, Longdan Xiegan pills, Qingkailing soft capsules, Niuhuang Shangqing soft capsules and Zhizi Jinhua pills [10]. On the other hand, in recent years, there have been many new findings on the hepatotoxicity and nephrotoxicity of the natural product [11,12], which may limit its usage as a candidate drug with good performance in some diseases.

Although a large number of studies on GS have been carried out and a considerable amount of relevant literature has been reported, a comprehensive review that provides a complete profile on the phytochemistry, pharmacology, pharmacokinetics and toxicology of the active component is still lacking. In the present study, with the help of some scientific search engines and databases, including Google Scholar, Web of Science, Pubmed and Chinese National Knowledge Infrastructure (CNKI), we summarize the recent research progress of the above aspects. In addition, some further studies and future perspectives were also proposed. We hope that this review would prove helpful to researchers to ensure that the GS will have an effective and safe application.

## 2. Sources

To our knowledge, researchers isolated and identified GS from *Gardenia jasminoides* forma grandiflora (Lour.) Makino as an iridoid glycoside-type natural product in the 1960s [13]. Since then, this compound has been found in nearly 40 plants, the majority of which belong to the Rubiaceae (Table 1). Some of the plants are famous phytomedicines that have been used in the clinic for thousands of years in China and Southeast Asia, such as *G. jasminoides* (Zhizi), *Rehmannia glutinosa* Libosch. (Dihuang), *Eucommia ulmoides* Oliv. (Duzhong) and *Achyranthes bidentata* Blume (Niuxi). Among them, *G. jasminoides* is a fundamental plant source of GS with a considerable content (3.3–8.56%) found in the fruits of dry weight (DW) [14]. This iridoid glycoside has been found and isolated from various parts of this phytomedicine, including leaves, flowers, fruits and tubers. Furthermore, there were also several varieties of the species *G. jasminoides* containing GS, including *Gardenia jasminoides* cv. fortuneana Hara, *Gardenia jasminoides* forma grandiflora (Lour.) Makino and *Gardenia jasminoides* var. *radicans* (Thunb.) Makino. There were also some studies on the GS contents in other herbal medicines from different production areas, which report various amounts such as 0.2035–0.4381% (DW) for *Rehmannia glutinosa* Libosch. roots [15], 0.0699–0.1135% (DW) for *Scrophularia ningpoensis* Hemsl. roots [16], and 0.0173–0.5811% (DW) for *Eucommia ulmoides* Oliv. barks [17].

## 3. Natural Derivatives

**Figure 2:**
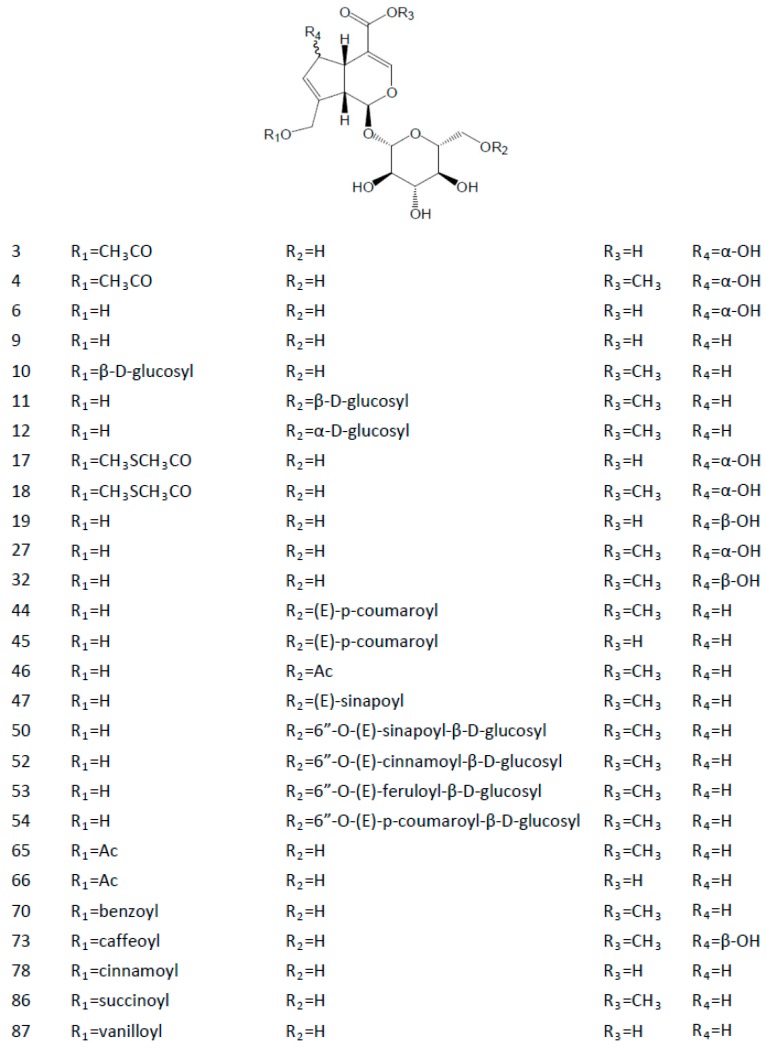

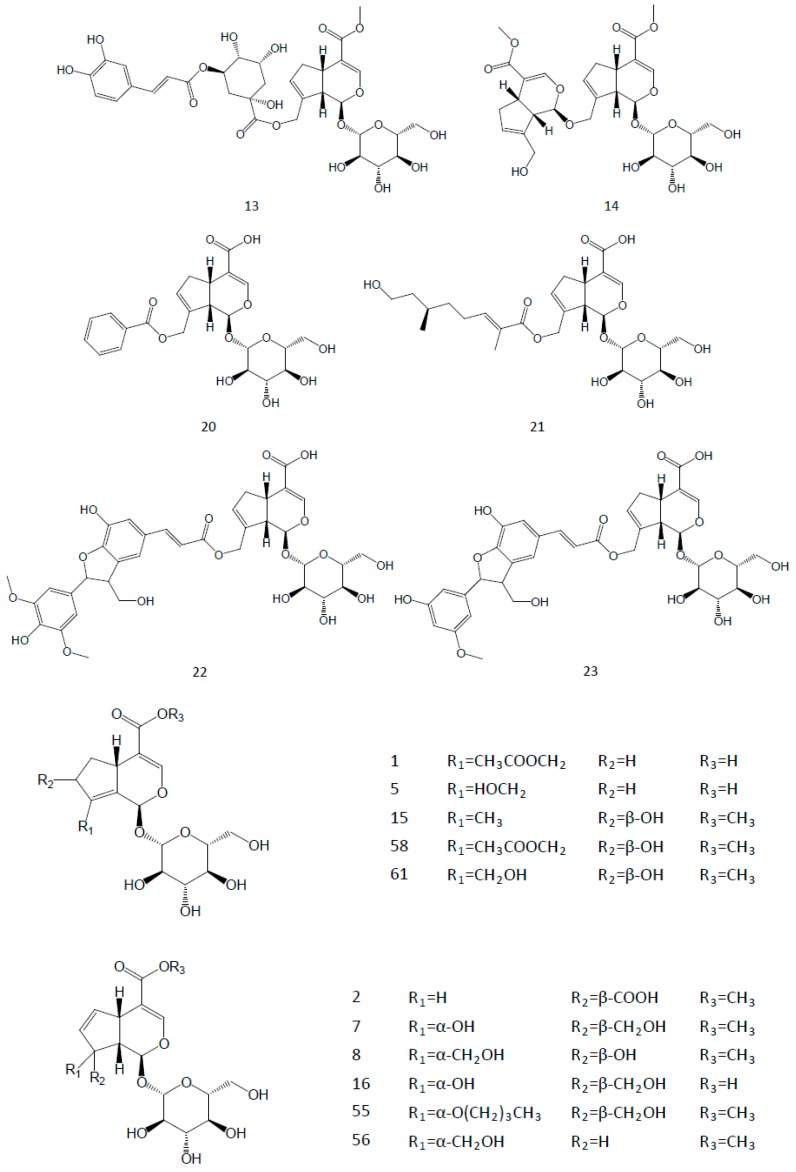
Chemical structures of some representative geniposide derivatives.

We know that approximately 90 derivatives have been found present in natural plants along with GS. Table 2 lists their names and Figure 2 shows the structures of the representative derivatives. The compounds listed here are all iridoid glycosides. Some are the substitution derivatives on the various positions of GS, such as geniposidic acid, scandoside and daphylloside. Some, including alpinoside, majoroside and monotropein, are derivatives of the structural isomers of GS with different double bond positions. Some are the biosynthetic products of GS, such as galioside, gardenoside, 6α-hydroxygeniposide and 6β-hydroxygeniposide [145].

As for its chemical structure, GS is regarded as an iridoid glycoside, namely genipin 1-*O*-β-d-glucopyranoside. From another point of view, this component is also regarded as a C11 methyl ester of geniposidic acid, which is a common derivative of GS found in Nature. Due to the bioactivities and high content in the phytomedicine, geniposidic acid was recorded as the chemical marker for the quality evaluation of Plantaginis Semen in the Chinese Pharmacopoeia [146]. Other derivatives commonly found in natural plants are 6α-hydroxygeniposide and 6β-hydroxygeniposide with their own derivatives. These two natural components are characterized by a α-OH or β-OH on the C6 position of GS. These hydroxyl groups and that on the C10 position are often esterified with some organic acids, such as acetic acid, ferulic acid, caffeic acid, cinnamic acid, succinic acid and *p*-coumaric acid. In addition, there are also some other derivatives with a C6–C7 double bond (e.g., gardenoside, galioside and monotropein) or a C8–C9 double bond (e.g., alpinoside and majoroside), which differ from the C7–C8 double bond of GS.

In terms of glycoside-moiety numbers, the above mentioned natural derivatives are mono-glycosides. Apart from these components, some diglycosides have also been discovered in the plants. For example, genipin gentiobioside is a diglycoside with a gentiobiose on the C1 position, while genipin isomaltoside has an isomaltose moiety on the C1 position. These two compounds are considered as 6′-*O*-β-d-glucosylgeniposide and 6′-*O*-α-d-glucosylgeniposide. The substitutions with different acids mentioned above are also found on the C6′ or C6′′ position of genipin gentiobioside. Furthermore, genipin 1,10-di-*O*-β-d-glucopyranoside is another diglycoside with two glucose molecules on C1 and C10, which is also known as 10-*O*-β-d-glucosylgeniposide.

## 4. Analytical Methods

Due to its higher separation efficiency, shorter analysis time and less sample consumption, liquid chromatography (LC) is considered as an accepted and effective analytical method to separate mixtures in the natural products research field. To determine the GS content in the different plant sources and medical preparations, high performance liquid chrpmatography (HPLC) or ultra performance liquid chromatography (UPLC) is usually employed, coupled with some detectors, such as an ultraviolet detector (HPLC-UV), diode array detector (HPLC-DAD) or evaporative light scattering detector (HPLC-ELSD) [9,10,14,147,148]. Since the GS content is much lower and some endogenous interfering substances are present in the plasma or other biological samples, a mass spectrometry detector (MSD) is much preferable, especially in multiple-reaction monitoring (MRM) mode when pharmacokinetic studies are involved [149,150,151].

Like HPLC, capillary electrophoresis (CE) is also characterized by high efficiency and high selectivity. Han et al. quantitatively analyzed GS along with other four phytochemicals in *Rehmannia glutinosa* Libosch. roots. Sodium borate (60 mM) was used as buffer solution (5% methanol, pH 9.5), the separation voltage was set at 20 kV, and the temperature was maintained at 20 °C [15]. Micellar electrokinetic chromatography (MEKC) is a CE mode used to separate either neutral or charged components. Along with other nine bioactive compounds, GS was successfully separated and determined in *Eucommia ulmoides* Oliv. barks by MEKC using 50 mM boric acid (pH 9.5) as the buffer solution, with 50 mM sodium dodecylsulfate and 4% 1-butanol [152].

Aside from the chromatographic methods mentioned above, the near-infrared spectroscopy (NIRS) technique has also been used for quantitative analysis of GS in *Gardenia jasminoides* Ellis fruits with a partial least squares (PLS) method in the spectral regions of 8660–7500 cm^−1^, 6650–5600 cm^−1^ and 4900–4000 cm^−1^ [153].

## 5. Pharmacology

With the rapid development of modern pharmacology, an increasing amount of evidence has emerged for the multiple bioactive functions of GS, including its anti-inflammatory, antitumor, anti-oxidative, neuroprotective, hepatoprotective and cholagogic effects.

### 5.1. Hepatoprotective and Cholagogic Effects

As a major component in *G. jasminoides* fruits, GS also has a hepatoprotective effect similar to the phytomedicine and has been considered as a potential drug for liver diseases. After a CCl_4_ challenge in mice in vivo, serum levels of alanine transaminase (ALT), aspartate transaminase (AST) and alkaline phosphatase (ALP) increased markedly, while in the liver homogenate, the level of glutathione (GSH) and activity of antioxidant enzymes (SOD and CAT) decreased significantly. Furthermore, deformability, irregular arrangement and rupture of hepatocytes were observed in the liver. These biochemical parameters and histopathological examination results indicated liver damage, which were all ameliorated by a peroral GS treatment of 400 mg/kg [154]. In terms of liver damage induced by *Tripterygium* glycosides, GS (i.g.; 20, 40, 80 mg/kg) also had protective effects in vivo, which may be involved in alleviating oxidative stress and inflammationin in addition to facilitating tissue repair and regeneration [155]. Furthermore, GS (i.g.; 20, 40, 80 mg/kg) could ameliorate alcohol-induced oxidative stress damage in the liver in vivo through upregulating the expression of the main antioxidant enzymes, including GSH, glutathione-*S*-transferase (GST), glutathione peroxidase (GPx), copper-and zinc-containing superoxide dismutase (CuZn-SOD) as well as catalase (CAT) [156]. Liver fibrosis is known to cause the destruction of the hepatic parenchyma and the liver structure. GS (20 μM) was able to inhibit epithelial-mesenchymal transition (EMT), which was induced by transforming growth factor β1 (TGF-β1) in hepatic fibrosis. This in vitro effect may be related to the inhibition of the TGFβ/Smad and external signal-regulated kinase/mitogen-activated protein kinase (ERK/MAPK) signaling pathways [157].

The homeostasis of bile acids between uptake, efflux and biosynthesis is essential. Once the balance is broken, cholestasis will occur and result in damage to the liver. In a related pharmacological investigation, α-naphthylisothiocyanate (ANIT)-induced rats were used to characterize the effect of GS on this disorder. Subsequently, associated with some regulation of enzymes and transporters contributing to the homeostasis of bile acids, such as organic anion transporting polypeptide 2, bile acids export pump and organic solute transporter β, the active component (i.g.; 25, 50, 100 mg/kg) was observed to reduce the uptake and biosynthesis of bile acids; to increase canalicular secretion; and to downregulate bile acids in plasma in vivo [5]. In another study, GS of 50 mg/kg (i.g.) also inhibited ANIT-induced cholestasis and liver damage in ICR mice in vivo, which was related to the downregulation of signal transducers and activators of transcription-3 (STAT3) and nuclear factor-κB (NF-κB) signaling [158].

### 5.2. Anti-Inflammation

In an anti-inflammatory study, the mouse mastitis model and the primary mouse mammary epithelial cells, both induced by lipopolysaccharide (LPS), were used to investigate the anti-mastitis effect of GS. The results suggested that GS (2.5, 5, 10 mg/kg in vivo; 25, 50, 100 μg/mL in vitro) could alleviate mammary gland apoptosis through the modulation of Toll-like receptor 4 (TLR4) and apoptosis-related factors, such as p53, Bax, BcI-2 and Caspase-3 [159]. In another study in vivo, 2,4,6-trinitrobenzene sulfonic acid (TNBS)-induced rat ulcerative colitis and LPS-infected Caco-2 cells were employed to evaluate the anti-inflammatory effect of GS. As a result, the iridoid glycoside was found to regulate abnormal NF-κB, cyclooxygenase-2, inducible nitric oxide synthase (iNOS), myosin light chain kinase (MLCK) protein expression and tight junction protein (occludin and zonula occludens-1) expression; as well as activating 5’-AMP-activated protein kinase (AMPK) phosphorylation. It was concluded that GS (i.g.; 25, 50 mg/kg) could ameliorate inflammation and modulate barrier dysfunction via the activation of the AMPK pathway [1].

As for acute lung injury (ALI), GS also showed good protective effect performance. In LPS-induced ALI mice in vivo, the natural product (i.p.; 20, 40, 80 mg/kg) inhibited the pathological changes in lung tissue, including alveolar wall changes, alveolar hemorrhage and neutrophil infiltration; reduced inflammatory cells and total protein concentration in the bronchoalveolar lavage fluid; and finally regulated inflammatory mediators, such as tumor necrosis factor-α (TNF-α), IL-6 and IL-10 [160].

### 5.3. Anti-Diabetic Effect

In China, as early as in the Tang Dynasty, *G. jasminoides* fruits were used in the clinic for treatment of “*Xiaoke*” (Type 2 diabetes), which was recorded in “*Yaoxinglun*”, a famous book on herbal medicines. In the early 1980s, Japanese researchers led by Kimura first revealed that GS derived from *G. jasminoides* fruits exhibited hypoglycemic actions in high sugar diet-fed rats in vivo at the dose of 100 and 300 mg/kg (i.g.) [161]. From then on, many studies have been carried out to examine this mechanism of action. In an in vitro study of HepG2 cells, GS (10, 100 μM) was found to suppress hepatic glucose production through activating AMPK, acetylcoenzyme A synthetase (ACC) and forkhead box class O1 (FoxO1) phosphorylation in addition to inhibiting phosphoenolpyruvate carboxykinase (PEPCK) and glucose-6-phosphatase (G6Pase) activities. The effects mentioned were related with the AMPK FoxO1 pathway to some extent, indicating the function of inhibiting hepatic gluconeogenesis in Type 2 diabetes [162]. Renal pathology is a common complication in diabetic patients. The levels of serum creatinine, blood urea nitrogen and urinary albumin are usually used to evaluate renal function. These indexes increased in streptozotocin-induced diabetic rats, which indicated renal dysfunction. Significant glomerular basement membrane thickening was also observed by histological examination. In the model rats pretreated with GS (i.g.; 50, 100 mg/kg), the abnormal structural and functional changes of kidney were all attenuated. The in vivo effects were concluded associated with an inhibition of NF-κB-mediated inflammatory response [163].

GS (10 μM) was shown to activate glucagon-like peptide 1 receptor (GLP-1R) and to improve glucose-stimulated insulin secretion (GSIS) in INS-1 pancreatic β cells in vitro [164]. The effect was found to be counteracted by preincubation with an inhibitor of phosphatidylinositol 3-phosphate kinase (PI3K), which suggested that a PI3K-dependent mechanism was perhaps involved and mediated with an increase of glucose transporter 2 (GLUT2) protein levels [165]. GS (10 μM) also exhibited the prevention in vitro against INS-1 cell damage induced by high-glucose through increasing heme oxygenase-1 (HO-1) and Bcl-2 protein levels; decreasing Bax protein level; as well as preventing caspase-3 cleavage. The findings indicated that AMPK played a fundamental role in the prevention of cell damage, which was confirmed by the effects of preincubation with an AMPK inhibitor and an AMPK activator [166]. Furthermore, GS of the same concentration as above could accelerate thioredoxin-interacting protein (Txnip) degradation in INS-1 pancreatic β-cells in vitro with high glucose (25 mM) treatment [167].

### 5.4. Neuroprotection

Synaptic and mitochondrial dysfunctions are commonly seen in the early stage of Alzheimer’s disease (AD). Amyloid-β (Aβ_1–42_) is able to induce axonal mitochondrial abnormalities and synaptic damage in cultured hippocampal neurons and model mice with AD. GS treatment (12.5, 25, 50 mg/kg in vivo, 2.5, 5, 10 μM in vitro) demonstrated the protection against the above dysfunction through attenuating axonal mitochondrial fragmentation, trafficking impairments and reactive oxygen species (ROS) elevation; protecting synaptic loss, abnormal spine density and morphology; and ameliorating the decrease in synapse-related proteins. The findings indicated GS as a potential drug to cease and prevent the early progression of AD [168].

A large body of evidence has shown that streptozotocin (STZ) is able to induce sporadic AD. However, intracerebral-ventricular (ICV) injection of GS (50 μM) was shown in vivo to prevent spatial learning deficit and tau phosphorylation in order to facilitate GSK3β (pS-9) expression and inhibit GSK3β (pY-216) expression, which were all induced by STZ. In terms of STZ-induced neural pathology, the active component could avert paired helical filament-like structures, vesicles accumulation in synaptic terminals, endoplasmic reticulum abnormalities and early stages of apoptosis [169]. In addition, GS could reduce Aβ production in addition to attenuating the corresponding neurotoxicity in neurons (10 μM) in vitro and amyloid precursor protein/presenilin 1 (APP/PS1) transgenic mice in vivo (i.g.; 40 mg/kg). As for the protective mechanism, the iridoid glycoside could induce the phosphorylation of Janus kinase 2 (JAK2) in addition to the signal transducers and activators of transcription 3 (STAT3). Furthermore, this glycoside can regulate the expression level of α- and β-secretase, which may be mediated with leptin signaling [170]. Furthermore, Aβ accumulation and cholinergic defects are considered to be related with learning and memory impairments. In cultured primary hippocampal neurons of middle-aged Alzheimer’s model mice in vivo, GS (i.p.; 12.5, 25, 50 mg/kg) inhibited the toxic effect of cholinergic deficits through increasing choline acetyltransferase (ChAT) activity and decreasing acetylcholinesterase (AChE) activity [4].

### 5.5. Immunomodulation

It is well known that rheumatoid arthritis (RA) is a chronic systemic disease and its pathogenesis is related to the imbalance of cellular and humoral immunity. In RA rats in vivo, GS (i.g.; 33, 66, 132 mg/kg) was observed to improve the cell proliferation of mesenteric lymph node lymphocytes (MLNLs); to decrease IL-6 and IL-17; to increase IL-4 and TGF-β1; and to attenuate histopathological changes. The rapidly accelerated fibrosarcoma/mitogen activated protein kinase kinase/extracellular signal-regulated kinase1/2(Raf/MEK/ERK 1/2) signaling pathway in MLNLs is considered to be involved in the mechanism of this bioactivity [171]. Allergic asthma, which is related to immune responses, is considered as a chronic inflammatory disease to the respiratory system with the cardinal pathophysiological symptoms, such as airway hyperresponsiveness, bronchoalveolar lavage eosinophilia, mucus hypersecretion;as well as increased levels of T-helper-2-associated cytokines, chemokines and serum ovalbumin (OVA)-specific immunoglobulin E (IgE). These changes were all found in OVA-challenged BALB/c mice. The pathological changes in vivo were attenuated by intraperitoneal injections of GS (80 mg/kg) and the effects were comparable to dexamethasone, a well-known anti-asthma drug [172].

### 5.6. Anti-Tumor

Irradiation at a low dosage is a reliable strategy to treat tumors although it can destroy the hematopoietic organs, including spleen, thymus and bone marrow, at high doses. Hsu et al. found that GS (i.p.; 500 mg/kg) was able to reduce undesirable damages of sub-lethal radiation to the hematologic tissue in vivo, which was beneficial to preventing tumors in the hematologic system [173]. Pretreatment of GS (0.2 or 1.0 μM) could also inhibit H_2_O_2_ and myeloperoxidase formation caused by 12-*O*-tetradecanoylphorbol-13-acetate (TPA) in addition to inhibiting TPA-induced skin tumor in female CD-1 mice in vivo [174].

### 5.7. Effects on Cardiocerebrovascular Diseases

Brain microvascular endothelial cell (BMEC) with oxygen–glucose deprivation (OGD) was a common model of cerebral ischemia in vitro. After the OGD challenge, the mRNA and protein expression of P2Y14 were upregulated. However, GS (33.2 μg/mL) in vivo was able to alleviate the abnormal tendency by suppressing the phosphorylation of RAF-1, mitogen activated protein kinase kinase1/2 (MEK1/2) and extracellular signal-regulated kinase 1/2 (ERK1/2); as well as declining the productions of IL-8, IL-1β and monocyte chemotactic protein 1 (MCP-1). The findings proved the potential of GS in treating cerebral ischemia in clinics [175]. Furthermore, GS (i.g.; 100 mg/kg) was proved to protect against atherosclerosis in vivo through inhibiting the development of atherosclerotic lesions; increasing Wnt1; decreasing dickkopf-related protein-1(DKK1) and NF-κB expression; reducing serum total cholesterol and low-density lipoprotein levels; and elevating the ratio of Wnt1/DKK1. Therefore, it was concluded that the protection of GS against atherosclerotic lesions were associated with enhancing Wnt1 and inhibiting DKK1 expression [176]. Another study on atherosclerosis was carried out in apolipoprotein E knockout (APOE-/-) mice. The findings indicated that GS (i.g.; 100 mg/kg) could promote the number and function of T-regulatory cells; in addition to decreasing plaque areas in the aorta, total cholesterol and low-density lipoprotein cholesterol in the serum, which contributed to stop the progression of atherosclerotic lesions in vivo [177].

As an agonist of GLP-1 receptor [164,178], GS also exerted inhibitory effects in cardiac hypertrophy. In mice with constriction of the transverse aorta, a functional decline in the heart was observed with a decrease in ejection fraction and fractional shortening, while the left ventricularinternal diastolic diameter increased significantly. All these morphological changes could be attenuated by GS treatment. In addition, the iridoid glycoside (i.g.; 25, 50 mg/kg) in vivo activated AMPKα and inhibited mTOR, ERK and ER stress in hypertrophic heart and in H9c2 cardiomyocytes. The protection and activation was mediated with GLP-1 receptor through the experiments of GLP-1 knockdown and blockade [179].

### 5.8. Other Effects

Apart from the actions mentioned above, GS also exhibited other effects, including anti-allergic, anti-depressive, anti-hyperuricemic, anti-oxidative and anti-thrombotic effects, which are shown in Table 3 [180,181,182,183,184,185,186,187].

## 6. Pharmacokinetics

With the rapid development in pharmacokinetics and further studies examining TCMs, an increasing amount of attention has been paid to natural products, especially bioactive ones, including GS.

### 6.1. Absorption

It is well known that the bioavailability of the drug can vary with different administration methods and GS is not an exception. To compare the bioavailabilities of GS, some Sprague Dawley (SD) rats were administered with this natural compound intragastrically (i.g.; 50 mg/kg), intramuscularly (i.m.; 8 mg/kg) and intranasally (i.n.; 8 mg/kg). The results demonstrated that the order of absolute bioavailability is i.m. (72.69%) > i.n. (49.54%) > i.g. (9.74%) [188]. GS was also found to penetrate the skin both in vivo and in vitro, allowing it to be quickly distributed in the subcutaneous tissue and blood after use of Shangyi Aerosol in mice [189]. To study the absorption mechanism of GS in Huanglian Jiedu Decoction, in vivo experiments and in vitro investigations, including intestinal perfusion and Caco-2 models, have been conducted. The results indicated that the GS absorption could be promoted by other co-existing compounds in the Chinese formula. In addition, GS was proved to be mainly absorbed by passive diffusion and to be a potential substance of P-glycoprotein in intestinal perfusion and Caco-2 models [151].

### 6.2. Distribution

In regard to tissue distribution after oral administration, the AUC_0→4h_ values of GS were calculated as the order of kidney > spleen > liver > heart > lung > brain [150].

### 6.3. Metabolism

In a metabolic profile experiment, normal male SD rats were orally administrated with GS at the dosage of 350 mg/kg. As a result, 33 metabolites were found and identified. Among them, there were 17, 31, six, 12, three, six, 12 and six metabolites detected in the plasma, urine, heart, liver, spleen, lung, kidney and brain, respectively. It was concluded that one fundamental metabolic pathway involved the hydrolysis of the hydroxyl groups on C-1, while the other was related with demethylation, methylation, cysteine conjugation, glycosylation and glucuronide conjugation [190]. In another study on the male rats with adjuvant arthritis, GS and the four metabolites, genipin (G1), the mono-glucuronide conjugate of genipin (G2), a cysteine conjugate ring-opened genipin (G3), an oxidation of G3 (G4) were detected and identified: GS, G1 and G2 in plasma; GS and G1 in mesenteric lymph node; only GS in liver and synovium; GS, G1, G3 and G4 in spleen; in addition to GS, G1, G2 and G3 in urine [191].

From the results of a pharmacokinetics study of GS, C_max_ was assayed as 0.68 ± 0.29 μg/mL at 0.44 ± 0.13 h and area under curve (AUC) was 1.46 ± 0.37 μg·h/mL [149]. To study the influence of gender on the pharmacokinetics of this natural product from *Eucommia ulmoides* Oliv. barks, some female and male rats were treated intragastrically with the extract of the plant. The pharmacokinetic parameters AUC_0→t_, AUC_0→∞_, *C*_max_, MRT_0→∞_ and *T*_1/2_ were quite different between the two sexes. It was observed that absorption was increased while the distribution and elimination were decreased in male rats compared with female rats, which showed gender influence on the metabolism of GS [192].

Apart from the pharmacokinetic study of GS alone in healthy rats, analogous studies of GS from some extracts or prescriptions in the model rats have been carried out. After the rats in the healthy group as well as Type 1 and Type 2 diabetic groups were orally administered with *G. jasminoides* extract, a longer *T*_max_, increased *C*_max_, MRT_0→∞_, AUC and decreased CL were observed in both diabetic groups while the values of *T*_1/2_ were similar among the three groups [193]. After i.v. infection of Reduning in the rats, the plasma concentration of GS reached its maximum around the administration time, before decreasing rapidly. The iridoid glycoside was eliminated quickly with a lower *T*_1/2_ (0.75 ± 0.04 h) [194].

### 6.4. Excretion

The excretion kinetics of GS was investigated with its concentration in urine. As for the volunteers in a clinic trial, the cumulative excretion amount of GS reached 70% as the prototype within 10 h after i.v. Naoxuening Injection. It was concluded that the majority of excretion of GS in human was via urine [195].

## 7. Toxicology

Although GS exerts many diverse biological activities, there are some studies examining its toxicity, which should be focused on by researchers. Hepatotoxicity was considered as the most fundamental issue of GS safety. An acute toxic study in rats revealed that GS of 574 mg/kg or above would result in hepatotoxicity associated with oxidative stress 24–48 h after oral administration. However, GS of 24.3 mg/kg or less would not cause hepatotoxicity even in the study of consecutive 90 days by oral administration [11]. As for the administration routes of GS, intranasal treatment was resulted in less hepatotoxicity than intravenous, intragastrical or intramuscular treatments [196]. The rats treated with GS had obvious hepatotoxicity, which did not show any significant differences between the SD rats and the Wistar rats of different ages. On the other hand, there was no obvious hepatotoxicity induced in the ICR mice [197].

Apart from the toxicity in normal rats, GS showed hepatotoxicity and nephrotoxicity in the rats with jaundice induced by ANIT. Alanine aminotransferase (ALT), aspartic transaminase (AST), alkaline phosphatase (ALP), total bilirubin (TBIL), blood urea nitrogen (BUN) and creatinine (CREA) activities in serum were increased after the model rats were treated with GS of 1.2 g/kg (i.g.). In addition, serious pathological damages in the liver and kidney of these rats were observed [12].

## 8. Conclusions and Future Perspectives

With the idea of “back to Nature”, the traditional phytomedicines and natural products have drawn attention from the field of medicine. As a traditional phytomedicine in East Asia, *G. jasminoides* fruits have been employed to “purge fire to relieve vexation, clear heart and drain dampness in addition to cooling the blood to remove toxins” in clinics for thousands of years. Since GS was first isolated in this herbal plant, a considerable number of scientists and researchers have started to focus on this active iridoid glycoside.

To our knowledge, the content of GS varies in the different plants and even in different parts of the same plant. To successfully perform pharmacological, toxicological and pharmacokinetic studies, it is necessary to prepare a considerable amount of this iridoid glycoside. Therefore, extraction–isolation–purification is the common and inevitable procedure to obtain the active compound. The following are the necessary aspects that researchers would have to consider in the further studies: (1) High efficiency is important in order to obtain a relatively high content and relatively low cost. Therefore, many plant origins and their parts will be tested to find the optimal ones for GS preparation; (2) It is important to save resources and making full use of them to avoid waste is preferable. For example, we should try to search for some other valuable extracts or natural compounds as byproducts when preparing GS from one plant or otherwise; (3) It is important to be environmentally friendly. During the preparation procedure of GS, we should choose less toxic or non-toxic solvents to reduce pollution as far as possible, which requires screening of preparation methods.

In some studies of different bioactivities, GS was proved as a GLP-1 receptor agonist to stimulate insulin secretion in pancreatic β-cells [2], exhibit antinociception [178] and protect against cardiac hypertrophy [179]. Apart from the abovementioned effects, GLP-1 was also found: (1) to decelerate gastric emptying by suppressing the gastrointestinal peristalsis and gastricacidsecretion [198]; (2) to increase intracellular cyclic adenosine monophosphate (cAMP) content, to accelerate calcium influx, to decrease the pyruvic acid content of the myocardium and to inhibit cardiomyocyte apoptosis [199,200,201]; and (3) to reduce the damage caused by stroke [202]. Nevertheless, few articles of GS have examined these physiological functions. Is GS able to show effects on the gastrointestinal system, cardiovascular system and nervous system through such mechanisms? It is worth investigating for the pharmacological researchers and the results may extend the range of GS bioactivities.

On the other hand, there has been concern about the potential toxicity of GS. It has even been used to induce liver damage in mouse models [203]. Despite needing to remain cautious regarding toxicity during the drug research and development stage, it is important to consider the contributing factors to this toxicity. Dosage may be one obvious issue. In the studies on GS toxicity, the experimental animals were often administered orally with this natural compound at several hundred mg/kg, which was several times the dosage used for disease treatment. Furthermore, it was reported that low-dose GS would not induce acute toxicity (28 mg/kg) [203] or chronic toxicity (24.3 mg/kg) [11]. Meanwhile, we also found that present pharmacological experiments were performed on model animals while the toxicological experiments were carried out on normal animals. Sometimes, the effective dosage in one pharmacological study was found to overlap the toxic dosage in another toxicological study. However, as we have known, the responses stimulated by the same dosage on model animals and normal ones are obviously different. Essentially, toxic experiments in the future should be also carried out in model animals, which could accurately reflect the actual situation of disease treatments. For each indication, it is better to try several dosages to determine either the effective dosage or the toxic dosage. Thus, the therapeutic window of the disease could be confirmed and the active compound could be employed safely. The next problem to resolve would be finding out which compounds are toxic in vivo after GS administration. As an iridoid glycoside with low bioavailability, GS converts into some metabolites in vivo, among which genipin, the aglycone of GS, is the primary one and has been proposed as the toxic compound. Nevertheless, this theory lacks enough support, especially from pharmacokinetic study on model animals at toxic dosages of GS. We also speculated that there may be significant differences between the pharmacokinetic profiles at high (toxic) dosage and low (therapeutic) dosages, which probably provides some clues of the toxic metabolites. Therefore, it is important to carry out the comprehensive absorption–distribution–metabolism–excretion–toxicity studies on both models and normal animals at different doses of GS with various administrations, with which we could explain the reason of inducing toxicity explicitly. On the other hand, the systematic structure–activity relationship (SAR) studies of GS derivatives should be included in the future with actual screening trials and virtual computer designs, which will guide the subsequent structural modification. With the findings, the effects of GS may be improved through increasing the bioavailability, reducing the toxicity and changing the solubility.

Taken together, as a bioactive natural product, GS may be developed as a candidate drug or a lead compound. However, a considerable amount of studies are still required. In this review, we have summarized the research on GS in various research fields in the recent years covering its phytochemistry, pharmacology, pharmacokinetics and toxicology. In spite of these valuable findings, some problems still remain unresolved, which have been proposed here and demand prompt solutions. There will be many interesting directions of this iridoid glycoside for either fundamental research or study of applications. Therefore, this paper will provide valuable background information to the researchers who are either interested in thorough investigation of the bioactivity of GS or to develop effective therapies based on this natural product.

## Figures and Tables

**Table 1:** Plants containing geniposide.

No.	Plant	Family	Part	References
1	*Achyranthes bidentata* Blume	Amarathaceae	roots	[18]
2	*Adina polycephala* Benth.	Rubiaceae	branches and stems	[19]
3	*Alibertia sessilis* (Vell.) K. Schum.	Rubiaceae	stems	[20]
4	*Biebersteinia heterostemon* Maxim.	Geraniaceae	whole plants	[21]
5	*Castilleja tenuiflora* Benth.	Orobanchaceae	whole plants	[22]
6	*Cistanche deserticola* Y. C. Ma	Orobanchaceae	stems	[23]
7	*Codonopsis pilosula* (Franch.) Nannf.	Campanulaceae	roots	[24]
8	*Cornus* *suecica* L.	Cornaceae	whole plants	[25]
9	*Cynanchum wilfordii* (Maxim.) Hemsl.	Asclepiadaceae	roots	[26]
10	*Dryopteris fragrans* (L.) Schott	Dryopteridaceae	whole plants	[27]
11	*Eucommia ulmoides* Oliv.	Eucommiaceae	barks	[28]
			leaves	[29]
12	*Gardenia jasminoides* cv. fortuneana Hara	Rubiaceae	leaves	[30]
13	*Gardenia jasminoides* forma grandiflora (Lour.) Makino	Rubiaceae	fruits	[31]
			leaves	[32]
14	*Gardenia jasminoides* var. *radicans* (Thunb.) Makino	Rubiaceae	fruits	[33]
15	*Gardenia jasminoides* Ellis	Rubiaceae	flowers	[34]
			fruits	[35]
			leaves	[36]
			tubers	[37]
16	*Gardenia sootepensis* Hutchins.	Rubiaceae	fruits	[38]
17	*Globularia davisiana* O. Schwarz	Globulariaceae	aerial parts	[39]
18	*Genipa americana* L.	Rubiaceae	fruits	[40]
19	*Hedyotis diffusa* Willd.	Rubiaceae	whole plants	[41]
20	*Hedyotis* *corymbosa* (Linn.) Lam.	Rubiaceae	whole plants	[42]
21	*Lantana camara* L.	Verbenaceae	roots	[43]
22	*Lippia alba* (Mill.) N.E. Brown	Verbenaceae		[44]
23	*Lonicera japonica* Thunb.	Caprifoliaceae	flower buds	[45]
24	*Oroxylum indicum* (L.) Kurz	Bignoniaceae	seeds	[46]
25	*Paederia scandens* (Lour.) Merrill	Rubiaceae	roots	[47]
26	*Randia spinosa* (Thunb.) Tirveng.	Rubiaceae	stems	[48]
27	*Rehmannia glutinosa* Libosch.	Scrophulariaceae	roots	[49]
28	*Rothmanniaglobosa* (Hochst.) Keay	Rubiaceae	fruits	[50]
29	*Scrophularia ningpoensis* Hemsl.	Scrophulariaceae	roots	[51]
30	*Stemona japonica* (Bl.) Miq.	Stemonaceae	roots	[52]
31	*Strychnos nux-vomica* L.	Loganiaceae	seeds	[53]
32	*Tinospora sagittata* var. *yunnanensis* (S. Y. Hu) H. S. Lo	Menispermaceae	roots	[54]
33	*Vangueria edulis* Vahl	Rubiaceae	flowers and leaves	[55]
34	*Vitex cannabifolia* Sieb. et Zucc.	Verbenaceae	fruits	[56]

**Table 2:** Natural derivatives of geniposide.

No.	Compounds	Sources
**1**	Alpinoside	*Globularia alypum* L. leaves [57], *Globularia aphyllanthes* Crantz aerial parts [58], *Globularia cordifolia* L. underground parts [59], *Globularia davisiana* O. Schwarz aerial parts [39], *Globularia dumulosa* O. Schwarz aerial parts [60], *Globularia trichosantha* Fisch. & C. A. Meyer whole plants [61], *Plantago asiatica* L. seeds [62], *Plantago alpina* L. aerial parts [63] and *Veronica cymbalaria* Bodard. aerial parts [64]
**2**	Apodanthoside	*Dioecrescis erythroclada* (Kurz) Tirveng leaves and branches [65], *Tocoyena Formosa* (Cham. & Schltdl.) K. Schum. stems [66] and *Vangueria edulis* Vahl flowers and leaves [55]
**3**	Asperulosidic acid	*Borreria verticillata* (L.) G. Mey. root barks [67], *Diodia teres* Walter whole plants [68], *Eucommia ulmoides* Oliv. leaves [69], *Galium aegeum* (Stoj. & Kitan.) Ancev aerial parts [70], *Galium humifusum* Bieb. aerial parts [71], *Galium macedonicum* Krendl. aerial parts [70], *Galium melanantherum* Boiss aerial parts [72], *Galium mirum* Rech. Fil. aerial parts [70], *Galium rhodopeum* Velen. aerial parts [70], *Galium rivale* (Sibth. and Sm.) Griseb. aerial parts [73], *Galium verum* subsp. verum L. aerial parts [74], *Globularia aphyllanthes* Crantz aerial parts [58], *Globularia trichosantha* Fisch. & C. A. Meyer whole plants [61], *Hedyotis* *corymbosa* (Linn.) Lam. whole plants [42] and aerial plants [75], *Hedyotis tenelliflora* Blume whole plants [76], *Lasianthus acuminatissimus* Merr. roots [77], *Morinda citrifolia* L. fruits [78] and seeds [79], *Oldenlandia diffusa* Roxb. aerial parts [80], *Oldenlandia umbellata* L.aerial parts [81], *Paederia scandens* (Lour.) Merrill roots [47] and *Saprosma scortechinii* Bl. King & Gamble stems [82]
**4**	Daphylloside	*Asperula lilaciflora* Boissaerial parts [83], *Borreria verticillata* (L.) G. Mey. root barks [67], *Globularia aphyllanthes* Crantz aerial parts [58], *Galium aegeum* (Stoj. & Kitan.) Ancev aerial parts [70], *Galium humifusum* Bieb.aerial parts [71], *Galium macedonicum* Krendl. aerial parts [70], *Galium mirum* Rech. Fil. aerial parts [70], *Galium verum* subsp. verum L. aerial parts [74], *Hedyotis corymbosa* (Linn.) Lam.whole plants [84], *Hedyotis diffusa* Willd. whole plants [41], *Hedyotis tenelliflora* Blume leaves [85] and *Lasianthus wallichii* (Wight & Arn.) Wight leaves [86]
**5**	Deacetylalpinoside	*Globularia dumulosa* O. Schwarz aerial parts [60], *Globularia trichosantha* Fisch. & C. A. Meyer whole plants [61]
**6**	Deacetylasperulosidic acid	*Asperula lilaciflora* Boiss aerial parts [83], *Borreria verticillata* (L.) G. Mey. root barks [67], *Eucommia ulmoides* Oliv. leaves [69], *Jasminum officinale* L. var. *grandiflorum* buds [87], *Galium aegeum* (Stoj. & Kitan.) Ancev aerial parts [70], *Galium humifusum* Bieb. aerial parts [71], *Galium macedonicum* Krendl. aerial parts [70], *Galium melanantherum* Boiss aerial parts [72], *Galium mirum* Rech. Fil. aerial parts [70], *Galium rhodopeum* Velen. aerial parts [70], *Galium rivale* (Sibth. and Sm.) Griseb. aerial parts [73], *Galium verum* subsp. verum L. aerial parts [74], *Hedyotis corymbosa* (Linn.) Lam. whole plants [84], *Lasianthus acuminatissimus* Merr. roots [77], *Morinda citrifolia* L. fruits [78] and seeds [79], *Morinda officinalis* How roots [88], *Oldenlandia diffusa* Roxb. aerial parts [80], *Paederia scandens* (Lour.) Merrill var. Mairei (Leveille) Hara whole plants [89], *Saprosma scortechinii* Bl. King & Gamble leaves and stems [82] and *Serissa serissoides* (DC.) Druce whole plants [90]
**7**	Galioside	*Cornus canadensis* L.leaves [91], *Gardenia jasminoides* cv. fortuneana Hara leaves [30], *Hedyotis diffusa* Willd. whole plants [41], *Morinda officinalis* How roots [92], *Randia spinosa* (Thunb.) Tirveng. stems [48] and *Tocoyena Formosa* (Cham. & Schltdl.) K. Schum. stems [66]
**8**	Gardenoside	*Dicliptera chinensis* (L.) Juss. whole plants [93], *Dioecrescis erythroclada* (Kurz) Tirveng leaves and branches [65], *Gardenia jasminoides* Ellis flowers [34], fruits [94] and leaves [36], *Gardenia jasminoides* cv. fortuneana Hara leaves [30], *Genipa americana* L. fruits [40], *Hedyotis diffusa* Willd. whole plants [37] and *Randia spinosa* (Thunb.) Tirveng. stems [48]
**9**	Geniposidic aicd	*Adina polycephala* Benth. branches and stems [19], *Alibertia myrciifolia* Spruce ex K. Schum. aerial parts [95], *Alibertia sessilis* (Vell.) K. Schum. stems [20], *Asperula lutea* subsp. rigidula aerial parts [96], *Bellardia trixago* (L.) All. whole plants [97], *Canthium gilfillanii* leaves [98], *Castilleja tenuiflora* Benth. aerial parts [99], *Diodia teres* Walter whole plants [68], *Eremophila longifolia* F. Muell. leaves [100], *Eucommia ulmoides* Oliv. leaves [69,101], *Euphrasia pectinata* Ten. aerial parts [102], *Galium aegeum* (Stoj. & Kitan.) Ancev aerial parts [70], *Galium humifusum* Bieb. aerial parts [71], *Galium melanantherum* Boiss aerial parts [72], *Galium mirum* Rech. Fil. aerial parts [70], *Galium rhodopeum* Velen. aerial parts [70], *Galium rivale* (Sibth. and Sm.) Griseb. aerial parts [73], *Gardenia sootepensis* Hutchins. fruits [38], *Gardenia jasminoides* fruits [103], *Genipa americana* L. fruits [40], *Globularia trichosantha* Fisch. & C. A. Meyer whole plants [61], *Lantana montevidensis* (Spreng.) Briq. roots [104], *Morinda longissima* Y. Z. Ruan roots [105], *Oldenlandia diffusa* Roxb. aerial parts [80], *Pedicularis longiflora* Rudolph whole plants [106], *Pedicularis plicata* Maxim whole plants [107], *Pedicularis verticillata* L. whole plants [108], *Plantago alpina* L. aerial parts [63], *Plantago depressa* Willd whole plants [109], *Rehmannia glutinosa* Libosch. roots [49,110], *Scyphiphora hydrophyllacea* Gaertn. F. aerial parts [111] and stem barks [112], *Vangueria edulis* Vahl flowers and leaves [55], *Verbascum lasianthum* Boiss. ex Bentham flowers [113], *Veronica anagallis-aquatica* L. whole plants [114], *Veronica bellidioides* L. aerial parts [115] and *Veronica kellererii* aerial parts [115]
**10**	Genipin 1,10-di-*O*-β-d-glucopyranoside	*Gardenia jasminoides* Ellis flowers [34] and fruits [94] and *Genipa americana* L. fruits [40]
**11**	Genipin gentiobioside	*Gardenia jasminoides* Ellis flowers [34] and fruits [94,103], *Gardenia jasminoides* forma grandiflora (Lour.) Makino fruits [31] and leaves [32], *Genipa americana* L. fruits [40] and *Rehmannia glutinosa* Libosch roots [110]
**12**	Genipin isomaltoside	*Gardenia jasminoides* Ellis flowers and fruits [94]
**13**	Jasmigeniposide A	*Gardenia jasminoides* Ellis fruits [103]
**14**	Jasmigeniposide B	*Gardenia jasminoides* Ellis fruits [103]
**15**	Majoroside	*Plantago asiatica* L.seeds [62], *Plantago cornuti* Gouan L. aerial plants [64], *Plantago depressa* Willd whole plants [109] and *Platago major* L. aerial parts [116]
**16**	Monotropein	*Cornus canadensis* L. leaves [91], *Cornus suecica* L.whole plants [25], *Coussarea platyphylla* Müll. Arg. [117], *Damnacanthus officinarum* Huang roots [118], *Galium aegeum* (Stoj. & Kitan.) Ancev aerial parts [70], *Galium humifusum* Bieb.aerial parts [71], *Galium macedonicum* Krendl. aerial parts [70], *Galium melanantherum* Boiss aerial parts [72], *Galium mirum* Rech. Fil. aerial parts [70], *Galium rhodopeum* Velen. aerial parts [70], *Galium rivale* (Sibth. and Sm.) Griseb.aerial parts [73], *Galium verum* subsp. verum L. aerial parts [74], *Morinda officinalis* How roots [88], *Pyrola calliatha* H. Andres whole plants [119], *Pyrola decorate* leaves [120], *Pyrola elliptica* roots [121], *Pyrola japonica* whole plants [122], *Pyrola xinjiangensis* Y. L. Chou whole plants [123] and *Saprosma scortechinii* Bl. King & Gamble leaves [82]
**17**	Paederosidic acid	*Paederia pertomentosa* Merr. ex Li aerial parts [124], *Paederia scandens* (Lour.) Merrill roots [47] and stems [125], *Paederia scandens* (Lour.) Merrill var. Mairei (Leveille) Hara whole plants [89], *Saprosma fragrans* Beddome aerial parts [126], *Saprosma scortechinii* Bl. King & Gamble leaves and stems [82] and *Serissa serissoides* (DC.) Druce whole plants [90]
**18**	Paederosidic acid methyl ester	*Paederia scandens* (Lour.) Merrill roots [47] and stems [125]
**19**	Scandoside	*Asperula lutea* subsp. rigidula aerial parts [96], *Cornus canadensis* L. leaves [91], *Galium aegeum* (Stoj. & Kitan.) Ancev aerial parts [70], *Galium humifusum* Bieb. aerial parts [71], *Galium macedonicum* Krendl. aerial parts [70], *Galium melanantherum* Boiss aerial parts [72], *Galium mirum* Rech. Fil. aerial parts [70], *Galium rhodopeum* Velen. aerial parts [70], *Galium rivale* (Sibth. and Sm.) Griseb. aerial parts [73], *Globularia aphyllanthes* Crantz aerial parts [58], *Globularia trichosantha* Fisch. & C. A. Meyer whole plants [61], *Oldenlandia diffusa* Roxb. aerial parts [80], *Oldenlandia umbellata* L. aerial parts [81], *Paederia scandens* (Lour.) Merrill var. Mairei (Leveille) Hara whole plants [89] and *Saprosma scortechinii* Bl. King & Gamble stems [82]
**20**	Scyphiphorin A	*Hedyotis corymbosa* (Linn.) Lam. whole plants [127] and *Scyphiphora hydrophyllacea* Gaertn. F. stem barks [112]
**21**	Scyphiphorin B	*Scyphiphora hydrophyllacea* Gaertn. F. stem barks [112]
**22**	Scyphiphorin C	*Scyphiphora hydrophyllacea* Gaertn. F. stem barks [128]
**23**	Scyphiphorin D	*Scyphiphora hydrophyllacea* Gaertn. F. stem barks [128]
**24**	Theveside	*Lantana montevidensis* (Spreng.) Briq. roots [104] and *Lippia alba* leaves [129]
**25**	4′-*O*-β-d-Glucopyranosyl-geniposide	*Genipa americana* L. fruits [40]
**26**	4′′-*O*-(*E*)-*p*-Coumaroylgenipin gentiobioside	*Gardenia jasminoides* Ellis fruits [130]
**27**	6α-Hydroxygeniposide	*Adina polycephala* Benth. branches and stems [19], *Alibertia sessilis* (Vell.) K. Schum. stems [20], *Galium aegeum* (Stoj. & Kitan.) Ancev aerial parts [70], *Galium macedonicum* Krendl. aerial parts [70], *Galium melanantherum* Boiss aerial parts [72], *Galium verum* L. whole plants [131], *Galium verum* subsp. verum L. aerial parts [74], *Gardenia jasminoides* Ellis flowers [34], fruits [94] and tubers [37], *Gardenia jasminoides* cv. fortuneana Hara leaves [30], *Gardenia jasminoides* forma grandiflora (Lour.) Makino leaves [32], *Gardenia sootepensis* Hutchins. fruits [38], *Hedyotis corymbosa* (Linn.) Lam.whole plants [84], *Hedyotis diffusa* Willd. whole plants [41], *Hedyotis tenelliflora* Blume leaves [85], *Oldenlandia umbellata* L. aerial parts [81], *Paederia pertomentosa* Merr. ex Li aerial parts [124], *Paederia scandens* (Lour.) Merrill stems [125], *Pittosporum glabratum* Lindl. roots [132], *Plantago lagopus* L. aerial parts [133] and *Randia spinosa* (Thunb.) Tirveng. stems [48]
**28**	6α-Methoxygeniposide	*Gardenia jasminoides* Ellis fruits [94], *Hedyotis tenelliflora* Blume leaves [85]
**29**	6α-*n*-Butoxygeniposide	*Gardenia jasminoides* Ellis forma grandiflora (Lour.) Makino fruits [134]
**30**	6α-Methoxygeniposidic acid	*Hedyotis tenelliflora* Blume leaves [85]
**31**	6β-Ethoxygeniposide	*Gardenia jasminoides* Ellis flowers [34]
**32**	6β-Hydroxygeniposide	*Adina polycephala* Benth. branches and stems [19], *Alibertia sessilis* (Vell.) K. Schum.stems [20], *Biebersteinia heterostemon* Maxim.whole plants [21], *Borreria verticillata* root barks [67], *Cornus canadensis* L. leaves [91], *Gardenia jasminoides* cv. fortuneana Hara leaves [30], *Gardenia jasminoides* Ellis flowers [34] and fruits [94], *Gardenia jasminoides* forma grandiflora (Lour.) Makino leaves [32], *Gardenia sootepensis* Hutchins. fruits [38], *Hedyotis corymbosa* (Linn.) Lam. whole plants [42,79] and aerial plants [74], *Hedyotis diffusa* Willd. whole plants [41], *Hedyotis tenelliflora* Blume leaves [85] and roots [135], *Morinda citrifolia* L. fruits [78], *Oldenlandia diffusa* Roxb. aerial parts [80], *Oldenlandia umbellata* L. aerial parts [81], *Randia spinosa* (Thunb.) Tirveng. stems [48], *Vangueria edulis* Vahl flowers and leaves [55] and *Wendlandia formosana* Cowan leaves [136]
**33**	6β-Methoxygeniposide	*Hedyotis tenelliflora* Blume leaves [85] and *Wendlandia formosana* Cowan leaves [136]
**34**	6β-*n*-Butoxygeniposide	*Gardenia jasminoides* Ellis flowers [34] and *Gardenia jasminoides* Ellis forma grandiflora (Lour.) Makino fruits [134]
**35**	6β-*O*-β-d-Glucosylpaederosidic acid	*Paederia scandens* (Lour.) Merrill stems [125]
**36**	6β-*O*-(*E*)-*p*-methoxy-cinnamoylgeniposide	*Hedyotis diffusa* Willd.aerial parts [137,138] and whole plants [139]
**37**	6β-*O*-(*E*)-Feruloylgeniposide	*Hedyotis diffusa* Willd.aerial parts [137,138] and whole plants [139]
**38**	6β-*O*-(*E*)-*p*-Coumaroyl-geniposide	*Hedyotis diffusa* Willd.aerial parts [137,138] and whole plants [139]
**39**	6β-*O*-(*Z*)-Feruloylgeniposide	*Hedyotis diffusa* Willd.aerial parts [138]
**40**	6β-*O*-(*Z*)-*p*-Methoxycinnamoyl-geniposide	*Hedyotis diffusa* Willd.aerial parts [138] and whole plants [139]
**41**	6β-*O*-(*Z*)-*p*-Coumaroyl-geniposide	*Hedyotis diffusa* Willd.aerial parts [138] and whole plants [139]
**42**	6-*epi*-Paederosidic acid	*Saprosma scortechinii* Bl. King & Gamble stems [82]
**43**	6-*O*-Acetylscandoside	*Galium aegeum* (Stoj. & Kitan.) Ancev aerial parts [70]
**44**	6′-*O*-(*E*)-*p*-Coumaroylgeniposide	*Gardenia jasminoides* Ellis fruits [35,140]
**45**	6′-*O*-(*E*)-*p*-coumaroyl-geniposidic acid	*Gardenia jasminoides* Ellis fruits [35]
**46**	6′-*O*-Acetylgeniposide	*Gardenia jasminoides* Ellis fruits [35,140]
**47**	6′-*O*-(*E*)-Sinapoylgeniposide	*Gardenia jasminoides* Ellis fruits [35,130,140]
**48**	6’-*O*-(*E*)-caffeoyl-6α-hydroxygeniposide	*Gardenia jasminoides* Ellis fruits [130]
**49**	6′′-*O*-(*E*)-Sinapoylgenipin gentiobioside	*Gardenia jasminoides* Ellis fruits [35,101]
**50**	6′′-*O*-(*E*)-*p*-Coumaroylgenipin gentiobioside	*Gardenia jasminoides* Ellis fruits [35,103,130]
**51**	6′′-*O*-(*E*)-Cinnamoylgenipin gentiobioside	*Gardenia jasminoides* Ellis fruits [35]
**52**	6′′-*O*-(*E*)-Feruloylgenipin gentiobioside	*Gardenia jasminoides* Ellis fruits [103]
**53**	6′′-*O*-(*Z*)-*p*-Coumaroylgenipin gentiobioside	*Gardenia jasminoides* Ellis forma grandiflora (Lour.) Makino fruits [134]
**54**	8α-Butylgalioside	*Gardenia jasminoides* Ellis flowers [34]
**55**	8-*epi*-Apodantheroside	*Gardenia jasminoides* cv. fortuneana Hara leaves [30]
**56**	9-*epi*-6α-Methoxygeniposidic acid	*Morinda citrifolia* L. fruits [78]
**57**	10-Acetoxymajoroside	*Plantago cornuti* Gouan L. aerial plants [64], *Platago major* L. aerial parts [64]
**58**	10-Acetylscandoside	*Saprosma scortechinii* Bl. King & Gamble stems [82]
**59**	10-Deoxygeniposidic acid	*Scyphiphora hydrophyllacea* Gaertn. F. stem barks [128]
**60**	10-Hydroxymajoroside	*Plantago asiatica* L. seeds [62], *Plantago cornuti* Gouan L. aerial parts [141], *Plantago depressa* Willd whole plants [109], *Platago major* L. aerial parts [64]
**61**	10-Methoxyapodanthoside	*Vangueria edulis* Vahl flowers and leaves [55]
**62**	10-*O*-(4′′-*O*-Methylsuccinoyl)-geniposde	*Gardenia jasminoides* Ellis fruits [140]
**63**	10-*O*-Acetylgeniposide	*Gardenia jasminoides* Ellis fruits [35,140]
**64**	10-*O*-Acetylgeniposidic acid	*Plantago alpina* L. aerial parts [63] and *Scyphiphora hydrophyllacea* Gaertn. F. stem barks [128]
**65**	10-*O*-Acetylscandoside	*Eucommia ulmoides* Oliv. Leaves [69]
**66**	10-*O*-Benzoyl-6α-hydroxygeniposide	*Hedyotis corymbosa* (Linn.) Lam. whole plants [84,127] and aerial plants [75]
**67**	10-*O*-Benzoyl-6β-hydroxygeniposide	*Hedyotis corymbosa* (Linn.) Lam. whole plants [42,84,127] and aerial plants [75] and *Oldenlandia diffusa* Roxb. aerial parts [80]
**68**	10-*O*-Benzoylgeniposide	*Hedyotis corymbosa* (Linn.) Lam. whole plants [127]
**69**	10-*O*-Benzoyldeacetyl-asperulosidic acid	*Saprosma scortechinii* Bl. King & Gamble stems [82]
**70**	10-*O*-Benzoyl-6′-*O*-arabinosyl-6β-hydroxygeniposide	*Oldenlandia diffusa* Roxb. aerial parts [80]
**71**	10-*O*-Caffeoyl-6β-hydroxygeniposide	*Wendlandia formosana* Cowan leaves [136]
**72**	10-*O*-Caffeoyldaphylloside	*Wendlandia formosana* Cowan leaves [136]
**73**	10-*O*-(*E*)-3,4-Dimethoxycinnamoyl-geniposidic acid	*Leonotis nepetaefolia* (L.) R. Br. stems [142]
**74**	10-*O*-(*E*)-Caffeoylgeniposidic acid	*Avicennia marina* (Forssk.) Vierh. whole plants [143]
**75**	10-*O*-(*E*)-Caffeoyl-6α-hydroxygeniposide	*Gardenia jasminoides* Ellis tubers [37]
**76**	10-*O*-(*E*)-Cinnamoylgeniposidic acid	*Avicennia marina* (Forssk.) Vierh.whole plants [143]
**77**	10-*O*-(*E*)-*p*-Coumaroyl-6β-hydroxygeniposide	*Hedyotis corymbosa* (Linn.) Lam. whole plants [42,127] and aerial plants [75]
**78**	10-*O*-(*E*)-*p*-Coumaroyl-geniposidic acid	*Avicennia marina* (Forssk.) Vierh. whole plants [143]
**79**	10-*O*-*p*-Hydroxybenzoyl-6β-hydroxygeniposide	*Hedyotis corymbosa* (Linn.) Lam. whole plants [42,127] and aerial plants [75]
**80**	10-*O*-*p*-Hydroxybenzoyl-geniposidicacid	*Leonotis nepetaefolia* (L.) R. Br. stems [142]
**81**	10-*O*-*p*-Hydroxybenzoyl-geniposide	*Hedyotis corymbosa* (Linn.) Lam. whole plants [127]
**82**	10-*O*-Succinoylgeniposide	*Gardenia jasminoides* Ellis fruits [35]
**83**	10-*O*-Vanilloyl geniposidic acid	*Alibertia myrciifolia* Spruce ex K. Schum. aerial parts [95]
**84**	10-*O*-(*Z*)-*p*-Coumaroylmonotropein	*Vaccinium bracteatum* Thunb. branches and leaves [144]

**Table 3:** Other pharmacological effects of geniposide.

Function	Inducer	Model	Efficacy Evaluation	Reference
Anti-allergy (in vitro)	Compound 48/80	MC/9 cells	Inhibited histamine release	[180]
Anti-depression (in vivo)	STZ	Mice (i.g.; 50, 100 mg/kg)	Attenuated increased immobility time in FST, elevated BDNF levels, upregulated the mRNA expression of BDNF and TrkB	[181]
Anti-enterovirus (in vitro)	EV 71	Rd cells (1, 2, 3 mg/mL)	Inhibited both EV71 replication and viral IRES activity	[182]
Anti-hypopigmentation (in vitro)	Norepinephrine	Human epidermal melanocyte (1, 10,100 μM)	Upregulated c-kit production, abrogated the repression of tyrosinase activity and melanin production	[183]
Anti-hyperuricemia (in vivo)	Potassium oxonate	Mice (i.g.; 50, 100, 200 mg/kg)	Reduced SUA level, Elevated UUA level	[184]
Anti-osteoporosis (in vitro)	-	Osteoblast-like cells (MG-63, Saos-2), osteoclast (10^−1^–10^−5^ mg/mL)	Increased proliferation of osteoblast-like cells and proline incorporation activity, Inhibited osteoclast activity	[185]
Anti-oxidation (in vitro)	H_2_O_2_	HUVEC (12.5, 25, 50 μg/mL)	Increased the viability of injured cells, increased the activities of SOD, GSH-Px, NOS and NO production, decreased intracellular ROS level, reduced apoptosis rate, restored the potential of cell proliferation	[186]
Anti-thrombosis (in vivo)	Photochemical reaction	Male ICR mice (i.v.; 20, 40 mg/kg)	Prolonged the time required for thrombotic occlusion	[187]
	Collagen	Blood (7.7, 26, 77 μM)	Inhibited platelet aggregation and PLA_2_ activity.	

BDNF = brain-derived neurotrophic factor; EV = enterovirus; FST = forced swimming test; GSH-Px = glutathione peroxidase; HUVEC = human umbilical vein endothelial cell; IRES = internal ribosome entry site; NOS = nitric oxide synthase; PLA2 = phospholipase A_2_; SUA = serum uric acid; TrkB = tropomyosin-related kinase B; and UUA = urinary uric acid. - indicates no inducer.
